# Supplementary material for: Study of the Fatty Acid Profile of Milk in Different Sheep Breeds: Evaluation by Multivariate Factorial Analysis
Source: Animals (Basel). 2022 Mar 13;12(6):722. doi: 10.3390/ani12060722 (PMC8944521; doi:10.3390/ani12060722)
Supplement: Supplementary file 1 [file animals-12-00722-s001.zip › animals-1626521-supplementary.pdf]

# Study of the fatty acid profile of milk in different sheep breeds: evaluation by Multivariate Factorial Analysis

Giuseppe Conte, Valentino Palombo, Andrea Serra, Fabio Correddu, Mariasilvia D'Andrea, Nicolò Pietro Paolo Macciotta, and Marcello Mele

**Supplementary Table S1.** Feeding system details and feed composition

| | Massese | Comisana | Sarda (Tuscany) | Sarda (Sardinia) |
|---|---|---|---|---|
| Pasture Botanical composition | *Avena sativa*<br>*Lolium italicum*<br>*Trifolium repens* | *Avena sativa*<br>*Lolium italicum*<br>*Trifolium repens* | *Avena sativa*<br>*Lolium italicum*<br>*Trifolium repens* | *Lolium multiflorum*<br>*Hedisarum coronarium*<br>*Cychorium intybus* |
| *DM (%)* | *9.0* | *9.0* | *9.0* | *22.3* |
| *CP (%)* | *14.6* | *14.6* | *14.6* | *18.2* |
| *CF (%)* | *3.0* | *3.0* | *3.0* | *1.5* |
| *Ash (%)* | *10.9* | *10.9* | *10.9* | *10.4* |
| *NDF (%)* | *42.2* | *42.2* | *42.2* | *42.5* |
| *ADF (%)* | *25.6* | *25.6* | *25.6* | *25.2* |
| *ADL (%)* | *4.0* | *4.0* | *4.0* | *3.0* |
| Concentrate amount | ~ 800 g/head | ~ 800 g/head | ~ 800 g/head | ~ 800 g/head |
| *DM (%)* | *90.0* | *90.0* | *90.0* | *91.9* |
| *CP (%)* | *17.9* | *17.9* | *17.9* | *17.7* |
| *CF (%)* | *2.9* | *2.9* | *2.9* | *3.3* |
| *Ash (%)* | *7.8* | *7.8* | *7.8* | *6.9* |
| *NDF (%)* | *24.3* | *24.3* | *24.3* | *17.0* |
| *NSC (%)* | *32.7* | *32.7* | *32.7* | *31.8* |
| *NEL (Mcal kg-1 DM)* | *1.5* | *1.5* | *1.5* | *2.0* |
| *C16:0* | *17.8* | *17.8* | *17.8* | *15.3* |
| *C18:0* | *3.8* | *3.8* | *3.8* | *2.0* |
| *C18:1c9* | *21.9* | *21.9* | *21.9* | *23.0* |
| *C18:2c9c12* | *47.9* | *47.9* | *47.9* | *54.1* |
| *C18:3c9c12c15* | *4.3* | *4.3* | *4.3* | *3.5* |

Abbreviations: DM, dry matter; CP, crude protein; CF, crude fat; NDF, neutral detergent fibre; NSC, non structural carbohydrates; NEL, net energy lactation. Fatty acid values are given as g/100 g of total fatty acid.

**Supplementary Table S2.** Descriptive statistics for individual milk fatty acids (g/100g of total lipids) of Massese breed (n = 118)

| | Mean | SD | CV% | P5 | P95 | kurtosis |
|---|---|---|---|---|---|---|
| C4:0 | 3.28 | 0.60 | 18.33 | 2.32 | 4.73 | 0.70 |
| C6:0 | 1.55 | 0.48 | 30.76 | 0.83 | 2.27 | 0.07 |
| C8:0 | 2.01 | 0.50 | 24.76 | 1.28 | 2.21 | 0.07 |
| C10:0 | 6.04 | 1.70 | 28.11 | 3.32 | 8.64 | 0.35 |
| C10:1c9 | 0.08 | 0.05 | 58.12 | 0.03 | 0.19 | 4.85 |
| C12:0 | 3.68 | 0.91 | 24.73 | 2.31 | 4.94 | 0.72 |
| C13:0 | 0.09 | 0.04 | 47.91 | 0.04 | 0.19 | 2.72 |
| C14:0 | 9.51 | 1.92 | 20.21 | 6.28 | 12.36 | 0.41 |
| C14:0iso | 0.11 | 0.03 | 28.09 | 0.07 | 0.18 | 0.28 |
| C14:1c9 | 0.57 | 0.09 | 16.37 | 0.39 | 0.60 | 3.57 |
| C15:0 | 1.07 | 0.25 | 23.08 | 0.68 | 1.57 | 3.21 |
| C16:0iso | 0.25 | 0.05 | 20.73 | 0.16 | 0.34 | 0.69 |
| C16:0 | 22.44 | 3.01 | 13.38 | 17.56 | 26.97 | 0.47 |
| C16:1c9 | 0.86 | 0.41 | 48.12 | 0.27 | 1.59 | -0.63 |
| C18:0 | 10.11 | 4.31 | 42.57 | 6.56 | 13.12 | 34.58 |
| C18:1 t6-8 | 0.13 | 0.06 | 48.53 | 0.03 | 0.26 | 0.20 |
| C18:1t9 | 0.20 | 0.08 | 38.51 | 0.05 | 0.35 | 0.24 |
| C18:1t10 | 0.31 | 0.21 | 66.13 | 0.05 | 0.78 | 5.53 |
| C18:1t11 | 2.13 | 1.28 | 60.31 | 0.58 | 4.61 | -0.71 |
| C18:1c9 | 17.96 | 2.71 | 15.11 | 14.35 | 22.12 | 2.59 |
| C18:1t15 | 0.20 | 0.06 | 29.91 | 0.11 | 0.31 | 0.28 |
| C18:1c12 | 0.14 | 0.06 | 42.34 | 0.06 | 0.24 | 0.68 |
| C18:2n6 | 2.28 | 0.86 | 37.57 | 0.97 | 3.60 | 0.54 |
| C20:0 | 0.22 | 0.10 | 44.97 | 0.14 | 0.38 | 39.21 |
| C18:3n3 | 0.81 | 0.35 | 42.46 | 0.27 | 1.37 | -1.03 |
| C18:2c9t11 | 1.12 | 0.53 | 47.71 | 0.40 | 2.19 | -0.74 |
| C20:1c11 | 0.05 | 0.03 | 54.59 | 0.00 | 0.10 | 1.88 |
| C21:0 | 0.06 | 0.02 | 35.92 | 0.02 | 0.10 | 0.82 |
| C20:4n6 | 0.11 | 0.04 | 37.28 | 0.06 | 0.19 | 0.80 |
| C20:5n3 | 0.08 | 0.04 | 53.59 | 0.03 | 0.14 | -1.23 |
| C24:0 | 0.05 | 0.02 | 44.30 | 0.02 | 0.08 | 4.54 |
| C22:5n3 | 0.11 | 0.04 | 33.94 | 0.06 | 0.18 | 0.66 |
| C22:6n3 | 0.03 | 0.03 | 97.69 | 0.00 | 0.08 | 3.55 |

SD= Standard Deviation; CV%= coefficient of variation; P5= 5$^{th}$ percentile; P95= 95$^{th}$ percentile

**Supplementary Table S3.** Descriptive statistics for individual milk fatty acids (g/100g of total lipids) of Comisana breed (n = 303)

| | Mean | SD | CV% | P5 | P95 | kurtosis |
|---|---|---|---|---|---|---|
| C4:0 | 4.06 | 0.82 | 20.22 | 2.85 | 5.42 | 0.40 |
| C6:0 | 2.09 | 0.36 | 17.49 | 1.55 | 2.75 | 0.08 |
| C8:0 | 1.73 | 0.37 | 21.61 | 1.15 | 2.37 | 0.74 |
| C10:0 | 4.89 | 1.16 | 23.81 | 3.05 | 6.99 | 0.70 |
| C10:1c9 | 0.18 | 0.06 | 32.31 | 0.09 | 0.29 | -0.03 |
| C12:0 | 2.80 | 0.51 | 18.40 | 2.08 | 3.68 | 1.23 |
| C13:0 | 0.06 | 0.01 | 14.65 | 0.05 | 0.08 | 0.67 |
| C14:0 | 8.59 | 0.92 | 10.66 | 7.22 | 10.19 | 0.16 |
| C14:0iso | 0.20 | 0.03 | 15.95 | 0.15 | 0.24 | 1.24 |
| C14:1c9 | 0.21 | 0.05 | 24.25 | 0.14 | 0.32 | 0.38 |
| C15:0 | 1.48 | 0.14 | 9.28 | 1.25 | 1.71 | 0.32 |
| C16:0iso | 0.43 | 0.06 | 13.63 | 0.33 | 0.53 | -0.08 |
| C16:0 | 22.58 | 1.65 | 7.29 | 20.46 | 25.40 | 2.39 |
| C16:1c9 | 0.70 | 0.07 | 9.97 | 0.58 | 0.81 | -0.16 |
| C18:0 | 9.45 | 1.31 | 13.83 | 7.32 | 11.41 | 1.46 |
| C18:1 t6-8 | 0.23 | 0.04 | 16.42 | 0.17 | 0.30 | 0.54 |
| C18:1t9 | 0.26 | 0.03 | 12.52 | 0.21 | 0.31 | 0.09 |
| C18:1t10 | 0.42 | 0.06 | 14.78 | 0.32 | 0.53 | 0.59 |
| C18:1t11 | 1.94 | 0.42 | 21.37 | 1.32 | 2.72 | 0.17 |
| C18:1c9 | 20.79 | 2.43 | 11.70 | 17.22 | 25.66 | 0.55 |
| C18:1t15 | 0.38 | 0.04 | 10.46 | 0.31 | 0.44 | 1.65 |
| C18:1c12 | 0.31 | 0.03 | 10.46 | 0.26 | 0.37 | 1.65 |
| C18:2n6 | 2.56 | 0.27 | 10.53 | 2.11 | 3.05 | 0.02 |
| C20:0 | 0.34 | 0.05 | 15.34 | 0.26 | 0.43 | 0.70 |
| C18:3n3 | 1.21 | 0.16 | 12.97 | 0.97 | 1.46 | 1.44 |
| C18:2c9t11 | 1.51 | 0.35 | 23.53 | 1.00 | 2.17 | 1.21 |
| C20:1c11 | 0.03 | 0.01 | 54.80 | 0.01 | 0.05 | 4.37 |
| C21:0 | 0.09 | 0.04 | 43.34 | 0.01 | 0.13 | -0.23 |
| C20:4n6 | 0.22 | 0.04 | 18.00 | 0.16 | 0.29 | 0.78 |
| C20:5n3 | 0.08 | 0.02 | 19.07 | 0.06 | 0.11 | 0.36 |
| C24:0 | 0.07 | 0.02 | 25.03 | 0.04 | 0.09 | 0.97 |
| C22:5n3 | 0.20 | 0.03 | 15.34 | 0.15 | 0.25 | 0.94 |
| C22:6n3 | 0.11 | 0.03 | 24.88 | 0.07 | 0.16 | 1.18 |

SD= Standard Deviation; CV%= coefficient of variation; P5= 5$^{th}$ percentile; P95= 95$^{th}$ percentile

**Supplementary Table S4.** Descriptive statistics for individual milk fatty acids (g/100g of total lipids) of Sarda breed reared in Tuscany (n = 133)

| | Mean | SD | CV% | P5 | P95 | kurtosis |
|---|---|---|---|---|---|---|
| C4:0 | 2.87 | 0.28 | 9.71 | 2.49 | 3.39 | 1.26 |
| C6:0 | 2.03 | 0.36 | 17.80 | 1.44 | 2.65 | -0.63 |
| C8:0 | 1.80 | 0.49 | 27.06 | 1.07 | 2.56 | -0.47 |
| C10:0 | 5.27 | 1.99 | 37.72 | 2.51 | 8.80 | -0.24 |
| C10:1c9 | 0.20 | 0.04 | 19.68 | 0.14 | 0.26 | -1.05 |
| C12:0 | 2.94 | 0.83 | 28.03 | 1.82 | 4.32 | 1.06 |
| C13:0 | 0.06 | 0.01 | 24.10 | 0.04 | 0.08 | -0.20 |
| C14:0 | 8.93 | 1.47 | 16.41 | 6.59 | 11.47 | -0.33 |
| C14:0iso | 0.10 | 0.02 | 14.85 | 0.08 | 0.13 | -0.81 |
| C14:1c9 | 0.13 | 0.06 | 45.52 | 0.06 | 0.24 | 1.53 |
| C15:0 | 0.81 | 0.15 | 18.74 | 0.56 | 1.07 | -0.21 |
| C16:0iso | 0.21 | 0.08 | 40.46 | 0.13 | 0.28 | 39.04 |
| C16:0 | 20.70 | 3.72 | 17.95 | 14.88 | 26.54 | 0.00 |
| C16:1c9 | 0.41 | 0.06 | 15.78 | 0.32 | 0.53 | -0.62 |
| C18:0 | 10.32 | 2.31 | 22.43 | 6.46 | 14.99 | 0.47 |
| C18:1 t6-8 | 0.42 | 0.19 | 44.91 | 0.17 | 0.73 | -0.99 |
| C18:1t9 | 0.42 | 0.15 | 36.49 | 0.24 | 0.69 | -0.82 |
| C18:1t10 | 0.58 | 0.17 | 30.30 | 0.35 | 0.84 | 7.43 |
| C18:1t11 | 3.56 | 2.68 | 75.16 | 1.16 | 9.69 | 1.41 |
| C18:1c9 | 18.83 | 3.62 | 19.22 | 13.10 | 24.81 | 0.72 |
| C18:1t15 | 0.48 | 0.19 | 38.76 | 0.19 | 0.82 | -0.40 |
| C18:1c12 | 0.27 | 0.05 | 19.19 | 0.19 | 0.35 | -0.41 |
| C18:2n6 | 2.10 | 0.38 | 18.15 | 1.58 | 2.74 | 0.94 |
| C20:0 | 0.20 | 0.04 | 21.11 | 0.13 | 0.27 | -0.11 |
| C18:3n3 | 1.43 | 0.66 | 46.12 | 0.56 | 2.51 | -1.15 |
| C18:2c9t11 | 1.44 | 0.91 | 63.04 | 0.59 | 3.61 | 2.45 |
| C20:1c11 | 0.05 | 0.02 | 45.85 | 0.02 | 0.09 | 14.55 |
| C21:0 | 0.06 | 0.03 | 46.36 | 0.03 | 0.10 | 30.43 |
| C20:4n6 | 0.09 | 0.03 | 37.80 | 0.04 | 0.15 | -0.36 |
| C20:5n3 | 0.05 | 0.02 | 34.20 | 0.03 | 0.08 | 1.30 |
| C24:0 | 0.05 | 0.02 | 32.73 | 0.02 | 0.08 | -0.09 |
| C22:5n3 | 0.09 | 0.02 | 25.40 | 0.06 | 0.14 | 0.60 |
| C22:6n3 | 0.04 | 0.01 | 35.49 | 0.02 | 0.06 | 1.11 |

SD= Standard Deviation; CV%= coefficient of variation; P5= 5$^{th}$ percentile; P95= 95$^{th}$ percentile

**Supplementary Table S5.** Descriptive statistics for individual milk fatty acids (g/100g of total lipids) of Sarda breed reared in Sardinia (n = 298)

| | Mean | SD | CV% | P5 | P95 | kurtosis |
|---|---|---|---|---|---|---|
| C4:0 | 2.67 | 0.34 | 12.62 | 2.20 | 3.17 | 0.71 |
| C6:0 | 1.82 | 0.33 | 18.07 | 1.29 | 2.37 | -0.13 |
| C8:0 | 1.70 | 0.42 | 24.74 | 1.01 | 2.36 | -0.07 |
| C10:0 | 5.93 | 1.65 | 27.81 | 3.43 | 8.70 | 0.04 |
| C10:1c9 | 0.26 | 0.08 | 30.47 | 0.15 | 0.40 | 0.57 |
| C12:0 | 3.72 | 0.97 | 26.13 | 2.30 | 5.39 | 0.66 |
| C13:0 | 0.03 | 0.01 | 33.29 | 0.02 | 0.05 | 0.71 |
| C14:0 | 11.22 | 1.42 | 12.68 | 8.96 | 13.33 | 0.16 |
| C14:0iso | 0.12 | 0.04 | 32.89 | 0.07 | 0.20 | 0.95 |
| C14:1c9 | 0.19 | 0.07 | 37.83 | 0.09 | 0.33 | 1.19 |
| C15:0 | 1.18 | 0.18 | 15.10 | 0.86 | 1.43 | -0.03 |
| C16:0iso | 0.34 | 0.07 | 19.94 | 0.25 | 0.46 | 0.42 |
| C16:0 | 25.65 | 2.85 | 11.10 | 21.26 | 30.37 | 0.43 |
| C16:1c9 | 0.85 | 0.21 | 25.21 | 0.55 | 1.23 | 1.14 |
| C18:0 | 10.20 | 2.24 | 21.96 | 6.43 | 13.79 | 0.14 |
| C18:1 t6-8 | 0.22 | 0.11 | 49.23 | 0.13 | 0.44 | 3.25 |
| C18:1t9 | 0.26 | 0.09 | 32.63 | 0.19 | 0.44 | 3.04 |
| C18:1t10 | 0.37 | 0.22 | 59.01 | 0.19 | 0.82 | 3.41 |
| C18:1t11 | 2.22 | 1.05 | 47.39 | 0.78 | 4.12 | 0.68 |
| C18:1c9 | 16.35 | 3.12 | 19.11 | 10.99 | 21.49 | 0.03 |
| C18:1t15 | 0.56 | 0.10 | 17.50 | 0.43 | 0.76 | 0.68 |
| C18:1c12 | 0.29 | 0.13 | 42.44 | 0.17 | 0.53 | 2.26 |
| C18:2n6 | 1.93 | 0.46 | 23.96 | 1.28 | 2.67 | 0.76 |
| C20:0 | 0.29 | 0.10 | 33.76 | 0.15 | 0.47 | 0.72 |
| C18:3n3 | 0.96 | 0.55 | 57.82 | 0.38 | 2.11 | 1.53 |
| C18:2c9t11 | 1.07 | 0.50 | 46.29 | 0.45 | 1.98 | 1.15 |
| C20:1c11 | 0.03 | 0.01 | 41.12 | 0.02 | 0.05 | 4.17 |
| C21:0 | 0.11 | 0.03 | 27.91 | 0.06 | 0.16 | 0.08 |
| C20:4n6 | 0.11 | 0.04 | 35.10 | 0.06 | 0.18 | 0.89 |
| C20:5n3 | 0.06 | 0.02 | 28.15 | 0.04 | 0.10 | 0.30 |
| C24:0 | 0.08 | 0.03 | 41.56 | 0.03 | 0.13 | 0.70 |
| C22:5n3 | 0.12 | 0.03 | 25.43 | 0.07 | 0.17 | 0.18 |
| C22:6n3 | 0.04 | 0.01 | 36.59 | 0.02 | 0.06 | 1.56 |

SD= Standard Deviation; CV%= coefficient of variation; P5= 5$^{th}$ percentile; P95= 95$^{th}$ percentile
